# High-Performance Hydrogen-Selective Pd-Ag Membranes Modified with Pd-Pt Nanoparticles for Use in Steam Reforming Membrane Reactors

**DOI:** 10.3390/ijms242417403

**Published:** 2023-12-12

**Authors:** Iliya Petriev, Polina Pushankina, Georgy Andreev, Sergei Ivanin, Stepan Dzhimak

**Affiliations:** 1 Department of Physics, Kuban State University, Krasnodar 350040, Russiaivanin18071993@mail.ru (S.I.);; 2 Laboratory of Problems of Stable Isotope Spreading in Living Systems, Southern Scientific Centre of the Russian Academy of Sciences, Rostov-on-Don 344006, Russia

**Keywords:** hydrogen permeability, Pd membranes, nanostars, hollow nanoparticles, modified surface, water gas shift reaction, membrane reactors

## Abstract

A unique method for synthesizing a surface modifier for metallic hydrogen permeable membranes based on non-classic bimetallic pentagonally structured Pd-Pt nanoparticles was developed. It was found that nanoparticles had unique hollow structures. This significantly reduced the cost of their production due to the economical use of metal. According to the results of electrochemical studies, a synthesized bimetallic Pd-Pt/Pd-Ag modifier showed excellent catalytic activity (up to 60.72 mA cm^−2^), long-term stability, and resistance to CO_ads_ poisoning in the alkaline oxidation reaction of methanol. The membrane with the pentagonally structured Pd-Pt/Pd-Ag modifier showed the highest hydrogen permeation flux density, up to 27.3 mmol s^−1^ m^−2^. The obtained hydrogen flux density was two times higher than that for membranes with a classic Pd_black_/Pd-Ag modifier and an order of magnitude higher than that for an unmodified membrane. Since the rate of transcrystalline hydrogen transfer through a membrane increased, while the speed of transfer through defects remained unchanged, a one and a half times rise in selectivity of the developed Pd-Pt/Pd-Ag membranes was recorded, and it amounted to 3514. The achieved results were due to both the synergistic effect of the combination of Pd and Pt metals in the modifier composition and the large number of available catalytically active centers, which were present as a result of non-classic morphology with high-index facets. The specific faceting, defect structure, and unusual properties provide great opportunities for the application of nanoparticles in the areas of membrane reactors, electrocatalysis, and the petrochemical and hydrogen industries.

## 1. Introduction

The rapid development of the global economy is creating high demand for energy in almost all areas of industry [1,2,3,4]. However, the main source of energy today is still fossil fuels, the reserves of which are very limited [5,6,7,8]. A promising next-generation alternative energy source is hydrogen [9,10,11,12]. The key step between the production and application of hydrogen is its separation and purification [13]. The membrane separation method is widely used in many areas, including hydrogen energy, due to its simplicity, low cost, high energy efficiency, and environmental friendliness [14,15,16,17]. The need to design and produce membrane filters with increased hydrogen permeability and durability has formed an interest in exploring different materials with the best performance and hydrogen resistance. 

Palladium-based membranes are the most demanded due to their unique hydrogen selectivity [18,19,20,21]. Nevertheless, the use of pure palladium in reactions involving hydrogen leads to poisoning and rapid mechanical destruction as a result of its embrittlement [22,23]. The solution would be to alloy palladium with other metals such as Ag, Cu, Ru, or Au [24,25,26]. The addition of silver to the alloy improves the mechanical resistance of palladium-based membranes and provides optimal hydrogen permeability. In their work [27], A. Suzuki et al. analyzed the inverse temperature dependence of hydrogen permeability through membranes made of pure Pd and its alloys. It was found that the addition of Ag to Pd increased hydrogen solubility at a high temperature and suppressed the α→α′ phase transition. This provided a moderate increase in the PCT factor at a higher temperature and resulted in a continuous hydrogen permeability peak at a higher peak temperature. Q. Zhou et al. [28] studied the selectivity and permeability of membranes for hydrogen on the basis of binary Pd-Au and ternary Pd-Au-Ag alloys. According to their results, both an improvement in the selectivity and a significant increase in the hydrogen permeability up to 2.09 × 10^−1^ mol s^−1^ m^−2^ Pa^−1^ at 300 K are achieved when Ag is introduced in comparison with the Pd-Au alloy membrane.

Nevertheless, the problem of low and unstable permeability of palladium-containing membranes at low-temperature operation modes, where hydrogen transport is limited by surface processes, remains unsolved [29,30]. This is caused by an impure or an inactive metal membrane surface. Consequently, there is a kinetic inhibition of reaching a balance between molecular hydrogen in the gas phase and atomic absorbed hydrogen in the palladium phase at the metal–hydrogen system border [31]. This condition can be partially overcome by surface activation. Thus, N. Vicinanza et al. [32] studied the effect of three-stage heat treatment of Pd77%Ag23% membranes in air on their hydrogen permeability. It was found that hydrogen permeability of membranes increased after each stage, as the use of the air heat treatment technique directly affected the increase in the effective surface area of the membrane. Another way to activate the membrane surface is its modification with powdered catalytically active particles [33,34,35,36]. The most promising way is the application of nanoparticles, which have become widespread in many areas of science and industry due to the unusual physical and chemical properties of nanoparticles [37,38,39,40,41,42]. The application of a nanoparticle-based modifying layer significantly increases the working surface area of a membrane, thereby promoting a chemisorption center increase [43]. Platinum group metals can be such hydrogen chemisorbing powders.

Palladium and platinum are universal catalysts for many reactions and processes in hydrogen energy [44,45,46,47]. However, most reactions show structural sensitivity, i.e., activity and selectivity depend on controlling metal nanoparticles’ shape and size and the arrangement of atoms on a surface [48,49,50,51]. Thus, the addition of high-index facets to palladium-based nanoparticles enhances their catalytic activity toward reactions involving hydrogen [52,53]. Therefore, pentagonally structured nanoparticles with a big number of high-energy facets are of major interest. The application of a modifier based on such nanoparticles to the surface of a palladium-containing membrane can significantly increase the catalytic activity of the material and the hydrogen permeability in low-temperature operation mode [54]. That is, the key factors contributing to the high activity of the material in reactions are the adjustment of the morphology and structure of particles (presence of high index facets, defects, and undercoordinated atoms) and the addition of a secondary metal [55,56,57,58,59]. In the first case, the given morphology is able to increase significantly the working surface of the membrane with an increased number of chemisorption centers [60]. In the second case, the addition of a secondary metal can greatly enhance the reactivity and stability of the material [61,62,63,64]. Consequently, the aim of this work was to study the effect of the morphology and structure of bimetallic nanoparticles as a part of palladium-silver membrane modifiers on low-temperature membrane processes of deep hydrogen purification.

## 2. Results and Discussion

### 2.1. Morphology, Structure Features, and Preparation of Nanoparticles on Pd Basis

**Figure 1:**
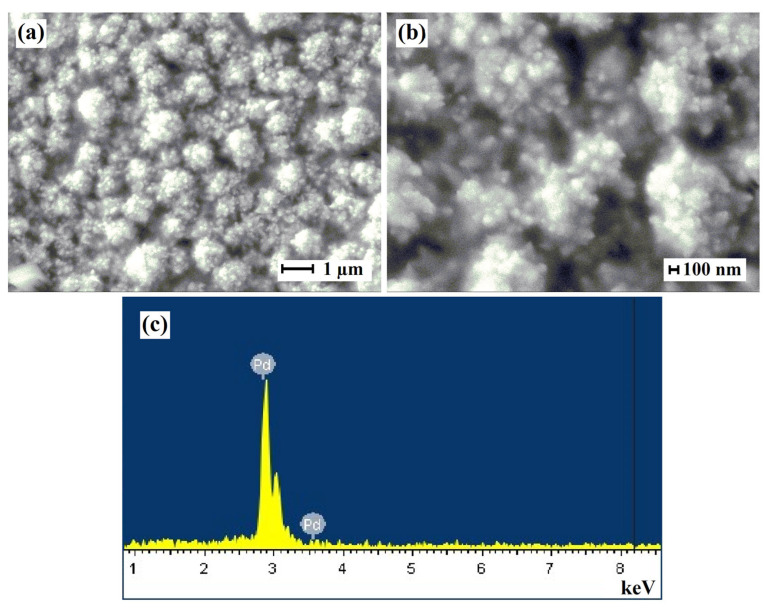
(**a,b**) SEM images of classic palladium nanoparticles at different magnifications. (**c**) EDS spectrum of the elemental composition of classic palladium nanoparticles.

Classic monometallic palladium nanoparticles were also obtained in the study. Microphotographs of the synthesized particles as a part of the modifier are presented in Figure 1a. The obtained particles had a classic energetically favorable spherical shape. The average size for 75% of the particles was about 80–110 nm. This classic particle type was deliberately obtained for further comparison with developed pentatwinned nanoparticles in catalytic and membrane applications. The results of the EDS analysis presented in Figure 1b showed 99.98% palladium content and few impurities in the synthesized classic palladium nanoparticles.

Non-classic bimetallic Pd-Pt nanoparticles as a part of the modifier with a fifth-order symmetry axis, which is unattainable in volumetric single crystals, were obtained in this study. The research concentrated on particle shaping and the combination of metals in their composition. The interest stemmed from the fact that catalytic reactions can occur more selectively on certain facets or due to the introduction of a secondary metal that alters reactivity. Palladium and platinum are relatively similar in many basic characteristics. Both metals have face-centered cubic lattices with negligible differences in the lattice parameter (Pd = 3890 Å, Pt = 3920 Å) and close standard reduction potentials. According to the density functional theory, platinum atoms occupy central positions, while palladium atoms concentrate on a surface during particle formation [65]. This can be explained by the higher surface energy and cohesion energy of platinum atoms.

Nevertheless, in the synthesis of nanoparticles, their deliberate design considering the faceted structure, homogeneity, and compositional control remains a challenge, especially for nanoparticles that are composed of catalytically favorable metal pairs. Therefore, a number of synthesis methods, which allow controlling the shape and composition of nanoparticles, have been developed in recent years [66]. The simplest and the most efficient method used in this work was the electrolytic deposition method. This method is unique because it provides additional control tools (voltage and current) along with classic tools for adjusting the shape and structure of particles (composition of the working solution and synthesis time). The developed method for the synthesis of non-classic pentatwinned particles combined several main distinctive features in comparison with the classic technique, which allowed the achievement of similar particle morphologies. First, a two-step current variation was applied in the deposition process. At the beginning, a sufficiently small current of 0.003 mA cm^−2^ was applied for a short period of time to promote the nucleation process. Such a step is important in the synthesis process as it is the nucleation shape, which underlies the nanoparticle, that can dictate self-assembly into larger architectures with new properties. Further, the current was significantly increased and maintained until the end of the synthesis. This allowed the directed growth of specific facets of the particle surface and giving them a certain shape. Second, the surfactant and halide ions were used as tools for adjusting and controlling the morphology. Properly selected surfactant concentration prevented particles from rounding during growth, preserving the inoculum geometry. Chloride in the working solution promoted oxidative etching, while bromide was responsible for selective passivation, stimulating the growth of facets with high Miller indexes. High-index facets exhibited much higher reactivity than low-index ones because they had a higher density of undercoordinated atoms located on steps and bends. These atoms had high reactivity, which was required for high catalytic activity. The choice of the electrolytic deposition method for particle synthesis was due to a number of advantages compared with colloidal methods. Additional simplified tools for customizing nanoparticle morphology and the simplicity of the method itself can be highlighted among these advantages. They made it possible to synthesize particles in a uniform thin layer immediately on the modified surface.

**Figure 2:**
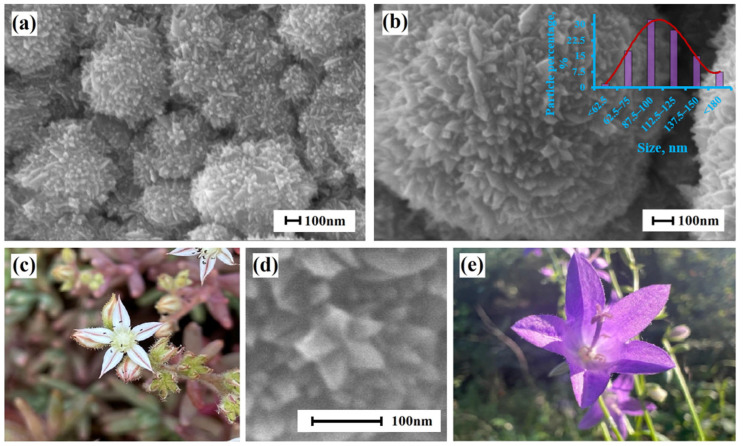
(**a**,**b**,**d**) SEM images of bimetallic Pd-Pt nanoparticles. The inset in (**b**) is the histogram of the Pd-Pt nanoparticle size distribution. (**c**,**e**) Photos of flowers that have fifth-fold symmetry. They are similar in shape to Pd-Pt nanoparticles.

Bimetallic Pd-Pt nanoparticles, synthesized in this work, had fifth-order rotational symmetry. Such symmetry can be observed in the flowers of many plants; however, it is not typical for objects of a non-living nature. Microphotographs of the surfaces of the obtained nanoparticles are shown in Figure 2. The particles had a five-pointed star shape with high-energy facets with a big number of undercoordinated atoms. The average size for 60% of the particles was in the range of 90–125 nm. 

**Figure 3:**
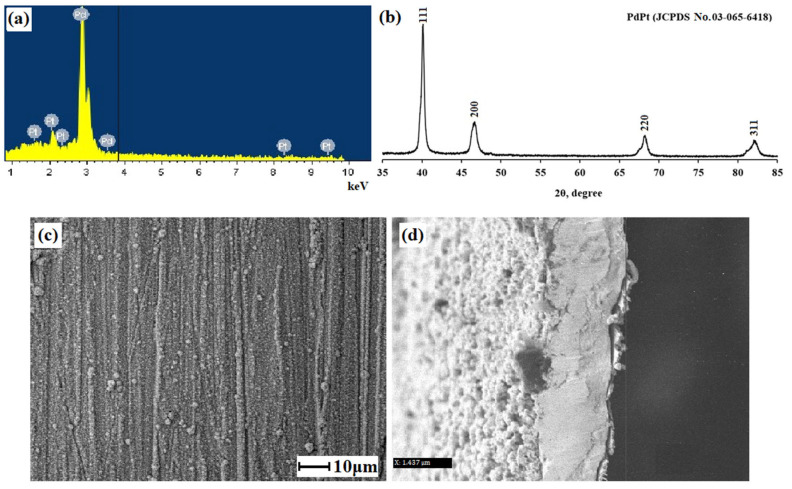
(**a**) EDS spectra of the elemental composition of synthesized bimetallic Pd-Pt nanoparticles. (**b**) X-ray diffraction spectrum of the synthesized modifier sample based on Pd-Pt nanoparticles. SEM images of the surface (**c**) and the section (**d**) of the membrane modified with pentagonally structured bimetallic Pd-Pt nanoparticles.

The obtained samples of films modified with pentagonally structured particles were characterized using EDS and XRD techniques. According to the EDS analysis (Figure 3a), the resulting bimetallic modifier contained 94.8% of palladium and 5.2% of platinum. Figure 3b shows the X-ray of the studied Pd-Pt alloy. The reflexes at 40.09, 46.56, 68.06, and 81.98 matched the (111), (200), (220), and (311) planes, respectively. Such an arrangement of peaks corresponded to the face-centered cubic lattice of the Pd-Pt alloy (JCPDS No. 03-065-6418). It should be noted that in the X-ray, reflections of the studied Pt-Pd alloy were located between single reflections of Pt (JCPDS-04-0802) [67] and Pd (JCPDS-46-1043) [68], which indicated the formation of a bimetallic Pt-Pd alloy with a face-centered cubic structure [69,70]. Figure 3c,d show SEM images of the surface and the section of the Pd-23%Ag membrane modified with pentagonally structured bimetallic Pd-Pt nanoparticles.

**Figure 4:**
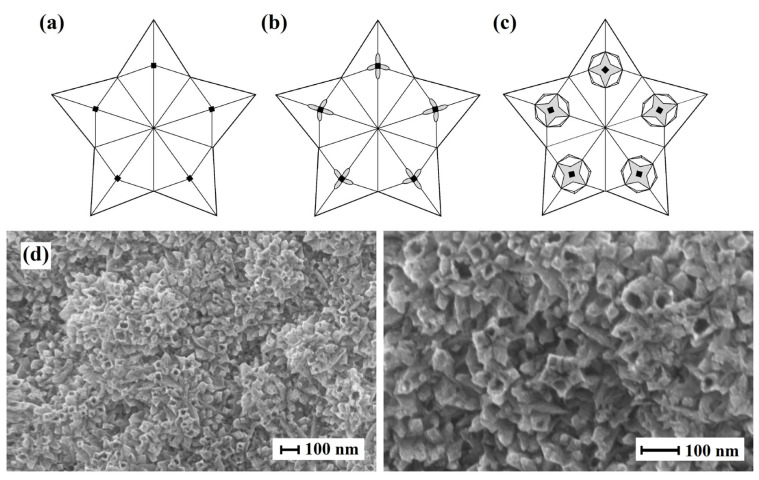
Scheme of crack formation in pentagonally structured nanoparticles during etching with concentrated HCl (35%): (**a**) initial state of particles with disclination lines marked with dots, (**b**) formation of separate cracks at twin boundaries, and (**c**) merging of cracks into a single star-shaped one. (**d**) SEM images of hollow Pd-Pt nanoparticles.

Moreover, it was found that pentagonal particles within the modifier were hollow. The obtained samples were weighed after the modifier was synthesized. However, according to the weighing results, the mass of the samples increased insignificantly. This raised a number of questions about the structure of the nanoparticles in the modifier composition. Chemical etching was used to determine structural features of the particle as the mechanical influence on particles of such a small size was a non-trivial task. The aim of the pentagonally structured particle etching experiment was the mechanical destruction of the outer shell of the particles; this should provide an understanding of the internal state of the structure, i.e., if the particles were solid or hollow inside This interest was motivated by the need to reduce costs and therefore reduce the amount of precious metal used in the production of such catalytic systems. During the etching process, the particle shell thinned, and multiple explosion-like ruptures appeared. It was confirmed with electron microscopy (Figure 4). Etching was performed with hydrochloric acid according to the mechanism that was described in another paper [71] for decahedral and icosahedral particles. This made it possible to speak about a similar destruction mechanism. The centers of destruction of pentagonal particles were the points of intersection of twin boundaries and disclinations on a particle surface. In other words, these were the points of the maximum concentration of internal elastic stresses. Consequently, it can be assumed that the disclination content in electrolytically synthesized particles can lead to the formation of internal voids in them. This particle structure certainly has an economic benefit in terms of precious metal reduced consumption.

### 2.2. Catalytic Characteristics of the Bimetallic Pd-Pt Modifier on Pentatwinned Nanoparticles Basis

**Figure 5:**
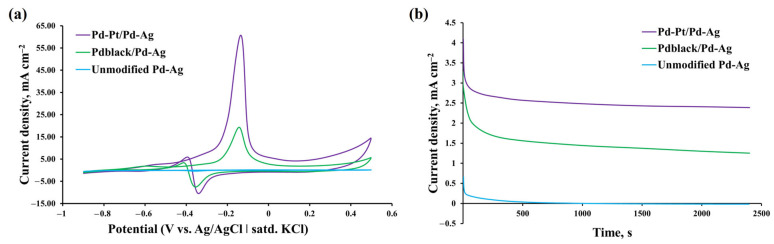
(**a**) Cyclic voltammetry curves of synthesized monometallic and bimetallic modifiers in alkaline oxidation reaction of methanol (1 M NaOH + 0.5 M CH_3_OH) at room temperature with a scanning potential rate of 50 mV s^−1^. (**b**) Chronoamperometric curves of synthesized monometallic and bimetallic modifiers in alkaline oxidation reaction of methanol (1 M NaOH + 0.5 M CH_3_OH) at a fixed potential of −0.3 V.

Electrochemical measurements of the obtained bimetallic pentagonally structured Pd-Pt/Pd-Ag modifier were carried out with cyclic voltammetry in alkaline methanol solution to assess the catalytic properties. They were also compared with the measurements for the classic Pd_black_/Pd-Ag modifier and the unmodified electrode. Scanning was performed at potentials from −0.9 V to 0.5 V toward Ag/AgCl (saturated KCl) at a scan speed of 50 mV s^−1^ at room temperature. Measurements for each sample were made for one hundred cycles. The 30th cycles, which were the highest ones, are shown in Figure 5a. All the studied samples showed similar trends, namely, a high current density peak around −0.1 V at anodic sweep (I_f_), which was caused by the oxidation of methanol. In addition, another peak around −0.4 V at cathodic sweep (I_b_) was observed for all samples, related to the accumulation of residual carbonaceous particles, which were produced during anodic sweep. However, the forward peak current density and reverse peak current density for the electrode with the bimetallic pentagonally structured Pd-Pt/Pd-Ag modifier were the highest and were approximately 60.72 mA cm^−2^ and 5.89 mA cm^−2^, respectively. The achieved values were three times higher than those for the electrode with the classic Pd_black_/Pd-Ag modifier, 19.28 mA cm^−2^ and 3.37 mA cm^−2^, respectively, and more than two orders of magnitude higher in comparison with the unmodified electrode. The observed improvement in the characteristics of the pentagonally structured Pd-Pt/Pd-Ag modifier in the methanol oxidation reaction can be explained with a bifunctional mechanism [72,73,74].

The process of methanol electrooxidation with electrodes modified with Pd-Pt particles can be described with the following stages [75]:

PdPt + CH_3_OH → PdPt-CO_ads_ + 4H^+^ + 4e^−^ (1)

PdPt + H_2_O → PdPt-OH_ads_ + H^+^ + e^−^ (2)

PdPt-CO_ads_ + PdPt-OH_ads_ → CO_2_ + 2PdPt + H^+^ + e^−^ (3)

According to (1), intermediate carbon monoxide forms (CO) were produced during the methanol oxidation reaction and then further adsorbed on the surface of the Pd-Pt modifier (PdPt-CO_ads_). The emerged CO_ads_ blocked the surface of the Pd-Pt modifier, thereby inhibiting the continuous oxidation process of methanol. PdPt-CO_ads_ can be oxidized by hydroxyl group (OH) with carbon dioxide (CO_2_) formation. 

In the synthesized bimetallic modifier, platinum was responsible for the chemisorption of methanol, and palladium was responsible for oxidation of water particles. Platinum adsorbed carbon intermediate compounds such as CO_ads_, and palladium adsorbed its counter intermediate compounds such as OH_ads_, i.e., it catalyzed the dehydrogenation of water molecules. In the bimetallic PdPt modifier, the d-zone centers of platinum shifted downward, and the Pt-CO_ads_ bond became weaker. At the same time, Pd-OH_ads_ reacted with it and eventually led to the formation of CO_2_. The reaction between Pt-CO_ads_ and Pd-OH_ads_ led to the removal of strongly adsorbed CO_ads_ particles on active centers. The strong bonding between Pd-Pt atoms can also reduce the coordination between the Pt and –CO_ads_ bonds, thereby destroying the Pt-CO_ads_. These synergistic effects significantly improved the overall characteristics of the Pd-Pt/Pd-Ag modifier. The enhanced electrocatalytic performance of the pentagonally structured Pd-Pt/Pd-Ag modifier in the methanol oxidation reaction can also be explained with the high density of atomic steps, protrusions, and fractures on high-index facets.

The resistance of catalytic coatings to CO_ads_ poisoning is usually assessed via the ratio of forward (I_f_) to reverse (I_b_) current density peaks [76]. In comparison with the classic Pd_black_/Pd-Ag modifier (5.7), the pentagonally structured Pd-Pt/Pd-Ag modifier showed higher values of the I_f_/I_b_ ratio—10.3. This indicated that poisoning particles were removed from the catalyst surface more efficiently and that the mutual contribution of palladium and platinum can significantly reduce CO poisoning during the reaction. The high value of the I_f_/I_b_ ratio implied that most of the intermediate carbonaceous particles CO_ads_ were oxidized to CO_2_ in the direct scan due to the presence of OH_ads_. 

Chronoamperometric tests of the pentagonally structured Pd-Pt/Pd-Ag modifier, the classic Pd_black_/Pd-Ag modifier, and the unmodified electrode were carried out to study the electrocatalytic stability, durability, and resistance to methanol oxidation at a fixed temperature. At the initial stage, the modified electrodes had high current values, which can be explained by a big number of active centers on the surface. Typically, methanol is continuously oxidized on the surface of the modifier at a fixed potential, and many intermediate adsorbed CO_ads_ particles also begin to accumulate on the surface during the methanol oxidation reaction. As can be seen from Figure 5b, the initial current of the pentagonally structured Pd-Pt/Pd-Ag modifier was significantly higher than that of the classic Pd_black_/Pd-Ag modifier and unmodified electrode. This was an indicator of a higher charging of the double layer [77]. However, a rapid drop of current up to 500 s was observed due to the formation of CO-like intermediates, which were adsorbed on the active centers of the catalysts. This prevented further oxidation of methanol [78]. It was recorded that the pentagonally structured Pd-Pt/Pd-Ag modifier demonstrated significantly higher current than that of the classic Pd_black_/Pd-Ag modifier and unmodified electrode over the entire time period, even though a current drop was observed. The final current density of the pentagonally structured Pd-Pt/Pd-Ag modifier was about 2.39 mA cm^−2^, which was higher than that of the classic Pd_black_/Pd-Ag modifier (1.25 mA cm^−2^) and the unmodified electrode (0.01 mA cm^−2^). In addition, the pentagonally structured Pd-Pt/Pd-Ag modifier had the lowest calculated current density reduction (41%) in comparison with the classic Pd_black_/Pd-Ag modifier (62%). This indicated better resistance of Pd-Pt/Pd-Ag to poisoning in the methanol oxidation reaction. The gradual decrease in the current over time was an indicator of the good anti-poisoning ability of the modifier [77]. The slower current decline, which was observed for the electrode with the pentagonally structured Pd-Pt/Pd-Ag modifier, indicated less accumulation of adsorbed CO_ads_ particles on the modifier surface. Therefore, it meant that the pentagonally structured Pd-Pt/Pd-Pd-Ag modifier showed superior electrocatalytic performance and better stability than the classic Pd_black_/Pd-Ag modifier and unmodified electrode toward the alkaline oxidation reaction of methanol. The activity level in the chronoamperometric measurements corresponded directly to the activity level in the cyclic voltammetry measurements. The obtained results can be due to the synergistic effect of the palladium-platinum alloy, which had superior poisoning resistance in comparison with monometallic palladium.

Thus, the bimetallic pentagonally structured Pd-Pt/Pd-Ag modifier, synthesized in this work, demonstrated excellent catalytic activity, long-term stability, and resistance to CO_ads_ poisoning in the alkaline oxidation reaction of methanol. The achieved results can be due to both the synergistic effect of the combination of palladium and platinum metals and the large number of available catalytically active centers, which are results of the non-classic morphology with high-index facets.

### 2.3. Diffusion and Selective Characteristics of Bimetallic Pd-Pt Modifier on Pentatwinned Nanoparticles Basis

**Figure 6:**
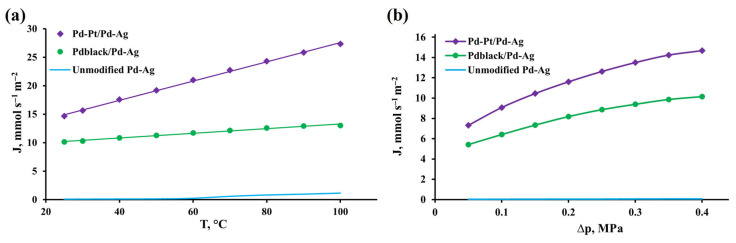
(**a**) Temperature dependence of hydrogen flux density at 0.4 MPa overpressure through membranes modified with developed monometallic and bimetallic nanoparticles. (**b**) Hydrogen flux density dependence on overpressure at 25 °C through membranes modified with developed monometallic and bimetallic nanoparticles.

The developed bimetallic pentagonally structured Pd-Pt/Pd-Ag modifier was studied in hydrogen transport processes to assess gas transport characteristics. The resulting characteristics were compared with those of the classic Pd_black_/Pd-Ag modifier and the unmodified membrane. In the first series of experiments, the diffusion characteristics of the obtained membranes were assessed in terms of hydrogen permeate flux density as a function of temperature in the range from 25 to 100 °C and a pressure of 0.4 MPa. The choice of this temperature range was based on the role and properties of the modifier to achieve permeability for palladium-based membranes even at room temperature. Figure 6a shows the temperature dependence of hydrogen flux density for membranes with the pentagonally structured modifier and the classic one. Data for the smooth unmodified Pd-Ag membrane are shown for comparison. It was evident that the flux of hydrogen permeating through the membranes increased with the rise of the permeation temperature. However, it should be noted that the permeation flux was not recorded up to a temperature of 50 °C for the unmodified membrane, while the modified membranes showed a hydrogen permeation flux density up to 14.7 mmol s^−1^ m^−2^ for Pd-Pt/Pd-Ag and 10.1 mmol s^−1^ m^−2^ for Pd_black_/Pd-Ag already at a temperature of 25 °C. The highest values of hydrogen permeation flux density at 100 °C were demonstrated by the membrane with a pentagonally structured Pd-Pt/Pd-Ag modifier, up to 27.3 mmol s^−1^ m^−2^. The obtained hydrogen flux density was two times higher than that for membranes with the classic Pd_black_/Pd-Ag modifier, up to 13 mmol s^−1^ m^−2^, and an order of magnitude higher than that for the unmodified membrane. To confirm the hypothesis of the influence of surface processes on permeability in the selected low-temperature range, the activation energy ($E_{A}$) was calculated using the Arrhenius equation [79]:

(4) $$P_{H_{2}}=P_{0}exp(\frac{{-E}_{A}}{RT})$$

where $P_{H_{2}}$ is the hydrogen permeability, $P_{0}$ is the pre-exponential multiplier, *R* is the universal gas constant, and *T* is the temperature. It is known from the literature [80] that *E_A_* values below 30 kJ mol^−1^ indicate a significant contribution of diffusion to the hydrogen transfer process, while surface phenomena require much higher activation energy up to 146 kJ mol^−1^. The *E_A_* for the developed membranes was estimated to be about 75 kJ mol^−1^ for the unmodified membrane and about 49 kJ mol^−1^ for the membrane with the pentagonally structured Pd-Pt/Pd-Ag modifier. Such results can be caused not only by the activation of the membrane surface, which accelerates surface limiting processes in the range of low temperatures (up to 100 °C), but also by the special morphology and structure of nanoparticles in the composition of the pentagonally structured modifier. Pentagonally structured particles, in contrast to classic spherical particles, had a large number of available catalytically active centers due to the presence of high-index high-energy facets. The synergetic effect of the favorable combination of palladium and platinum metals in the modifier also made a significant contribution. These conclusions were confirmed by electrochemical studies presented earlier, the results of which correlate closely with those of the gas transportation studies.

The second series of experiments was carried out to support the obtained data on the effect of the developed modifiers on the hydrogen permeability of palladium-containing membranes. During these experiments, the dependence of the permeate flux density as a function of overpressure in the range from 0.05 to 0.4 MPa and a temperature of 25 °C was studied. Figure 6b shows the pressure dependence of the hydrogen flux density for membranes with the pentagonally structured modifier and the classic one. A smooth unmodified Pd-Ag membrane is shown for comparison. In the conducted experiment, a similar dependence of the penetration flux density to that in the previous series of experiments was observed. Higher feed pressure determined higher driving force for hydrogen permeation, causing an increase in the density of flux permeated through the membrane. The membrane with the pentagonally structured Pd-Pt/Pd-Ag modifier had the highest hydrogen permeation flux density at 0.4 MPa, up to 14.7 mmol s^−1^ m^−2^. The achieved hydrogen flux density was 1.5 times higher than that for membranes with the classic Pd_black_/Pd-Ag modifier, up to 10.1 mmol s^−1^ m^−2^, and two order of magnitude higher than that for the unmodified membrane. However, the main point of this series of experiments was to identify the limiting stage of hydrogen transport for developed membrane materials. The hydrogen flux permeating through the membrane is expressed as follows [81]:

(5) $$J_{H_{2}}=\frac{P_{H_{2}}}{\delta }(p_{1}^{n}-p_{2}^{n})$$

where $J_{H_{2}}$ is the penetrating hydrogen flux, $P_{H_{2}}$ is the hydrogen permeability, *δ* is the membrane thickness, $p_{1}^{n}$ and $p_{2}^{n}$ are the partial pressure on the inlet and outlet sides of the membrane, respectively, and *n* is the pressure exponent. The exponent *n* can be from 0.5 to 1. At the boundary value *n* = 0.5, Equation (5) turns into the Sieverts–Fick law and points to a limitation of the transfer process by the diffusion of atomic hydrogen in the volume. In contrast, at the boundary value of *n* = 1, Equation (5) indicates that the transport process is limited by surface reactions, i.e., hydrogen dissociation/recombination takes a longer time and consequently consumes more energy than diffusion. According to the data presented in Figure 6b, the obtained permeate flux density for the unmodified membrane was easily approximated by a first-order line. The *n* value was 0.98, which meant that the transport process was completely limited by surface stages. This was confirmed by the activation energy, which was calculated above. For membranes with the pentagonally structured modifier, the exponent *n* was about 0.76, which indicated the control of hydrogen permeation flux by a combination of several mechanisms, namely, volumetric diffusion and surface processes. Moreover, it was confirmed by the evidently decreased activation energy in comparison with the unmodified membrane. The conducted series of experiments confirmed the acceleration of dissociative adsorption and recombinative desorption processes, which were limiting in the low-temperature range. Such acceleration was achieved by the activation of the membrane surface with the bimetallic pentagonally structured modifier with an increased number of reactive active centers toward hydrogen.

**Figure 7:**
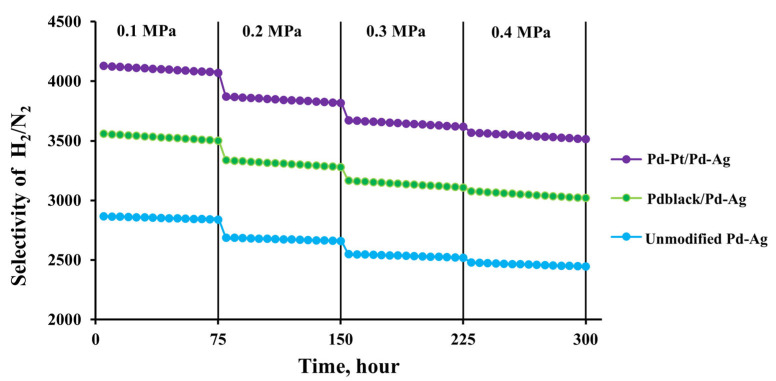
Selectivity dependence on overpressure at 25 °C through membranes modified with developed monometallic and bimetallic nanoparticles.

In the third series of experiments, hydrogen permeation and nitrogen leakage tests of the developed membranes with the bimetallic pentagonally structured Pd-Pt/Pd-Ag modifier were performed at 25 °C and a transmembrane pressure range from 0.1 to 0.4 MPa to assess selectivity. The results were compared with those for the classic Pd_black_/Pd-Ag modifier and the unmodified membrane. Figure 7 shows long-term permeability data for the membranes with the pentagonally structured modifier and the classic one for 300 h. A smooth unmodified Pd-Ag membrane is shown for comparison. According to the results, the developed membranes showed high selectivity over a long period of time. The membrane with the pentagonally structured Pd-Pt/Pd-Ag modifier demonstrated the highest H_2_/N_2_ selectivity at a pressure of 0.4 MPa, up to 3514. The achieved selectivity was 1.2 times higher than that for the membranes with the classic Pd_black_/Pd-Ag modifier, up to 3019, and was 1.5 times higher than that for the unmodified membrane. It can be seen from the data that the hydrogen permeation flux was increasing with each pressure rise and stabilized over time. During the whole penetration test, there was a slight drop in selectivity in the selected pressure range (0.1–0.4 MPa), but in numerical equivalent it could be considered as insignificant. It should be noted that the hydrogen flow was stabilized at a fixed pressure each time, and the nitrogen leakage did not increase either. This proved that the developed membranes demonstrated stability and resistance to pressure drops over a long period of time, as well as the absence of significant mechanical defects such as holes and seals.

## 3. Materials and Methods

### 3.1. Creation of a Membrane Basis, Methods of Its Modification, and Study of Its Surface Morphology

The membrane basis was thin palladium-silver foils, which were obtained by alloying palladium and silver components in an electric arc furnace. These metals in the form of ingots were immersed in a crucible and then alloyed in a chamber under a pressure of 0.05 MPa at a varying inverter current from 12 to 120 A. The resulting Pd-23%Ag alloy ingot was rolled on DRM–130 rollers (Durston, High Wycombe, UK) with intermediate annealings to a foil thickness of 20 μm.

**Figure 8:**
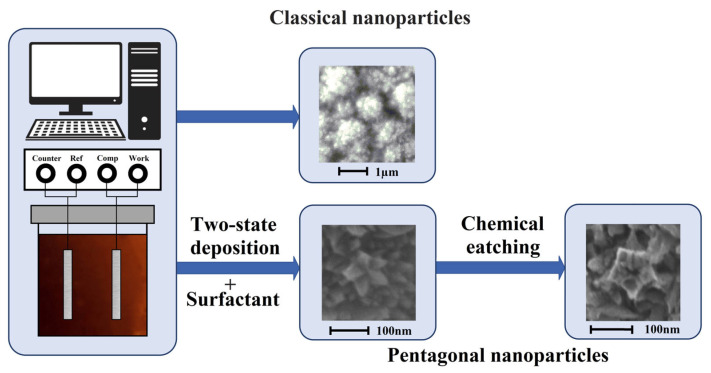
Schematic illustration of methods for synthesizing Pd and Pd-Pt nanoparticles.

The modification of the obtained Pd-23%Ag foils was carried out by electrolytic deposition in galvanostatic mode on a potentiostat-galvanostat P-40X (Electrochemical Instruments, Chernogolovka, Russia) in two ways (Figure 8). In the first classic method for the synthesis of monometallic palladium particles, the Pd-23%Ag foil was cleaned by washing in ethanol (96%) and degreasing in the 6 M NaOH solution. Afterward, the prepared foil was fixed in a working electrolytic cell and polarized first anodically in 0.1 M HCl, then cathodically in 0.05 M H_2_SO_4_. Polarization was carried out at a current density of 10–20 mA cm^−2^. After that, the cell was filled with the working solution of H_2_PdCl_4_ (2%) for further modification. Synthesis was carried out for 1.5–5 min at a current density of 5–6 mA cm^−2^. After deposition, the modified foil was washed with bidistillate.

In the second method for the synthesis of bimetallic pentatwinned Pd-Pt nanoparticles, the Pd-23%Ag foil was also cleaned according to the procedure, which was described in the first synthesis method. Subsequently, the prepared foil was moved to a working electrolytic cell, where it was anodically and cathodically polarized in 0.1 M HCl and 0.05 M H_2_SO_4_, respectively. Polarization was performed at a current density of 10–20 mA cm^−2^. Afterward, the cell was filled with the working solution, which contained C_16_H_36_BrN surfactant along with H_2_PdCl_4_ (2%). The palladium-platinum foil was used as an anode. During the synthesis process, a low current density of up to 0.003 mA cm^−2^ was set for a short period of time, 30–60 s. This stage was necessary for the nucleation process. Subsequently, the current density was increased to 0.25–0.3 mA cm^−2^, and particles were grown further for 3.5–10 min. After deposition, the modified foil was washed with bidistillate. All reagents were supplied by Sigma-Aldrich (St. Louis, MO, USA).

The morphology of the Pd-23%Ag modified foils was studied with electron microscopy on a JSM–7500F scanning electron microscope (JEOL, Tokyo, Japan). The elemental composition was monitored using an INCA (Oxford Instruments, Abingdon, UK) semiconductor energy dispersive attachment, which was a part of the JSM–7500F scanning electron microscope (JEOL, Tokyo, Japan).

### 3.2. Membrane Research in Catalytic and Gas Transportation Processes

The catalytic activity of the developed membrane materials was studied with cyclic voltammetry in the reaction of alkaline oxidation of methanol on the potentiostat-galvanostat P-40X (Electrochemical Instruments, Chernogolovka, Russia). The working solution consisted of 0.5 M methanol and 1 M NaOH. Measurements were performed at room temperature in the potential range from −0.9 V to + 0.5 V at a scan speed of 50 mV s^−1^. A three-electrode cell was used for the experiments. It consisted of a working electrode, which was made of developed membrane samples, a platinum counter electrode, and a glass Ag/AgCl reference electrode 

The long-term stability of the developed membrane materials was examined with chronoamperometry in the reaction of the alkaline oxidation of methanol on the potentiostat-galvanostat P-40X (Electrochemical Instruments, Chernogolovka, Russia). The working solution consisted of 0.5 M methanol and 1 M NaOH. Measurements were performed at room temperature at a constant potential of −0.3 V for 2400 s.

The gas diffusion characteristics and selectivity of the developed membrane materials were tested on a hydrogen permeability measurement unit according to the methodology, which was described extensively in another paper [82]. The examined membranes were hermetically sealed with copper gaskets and mounted in the chamber. Penetration tests were performed sequentially in hydrogen at various pressures up to 0.4 MPa and temperatures from 25 to 100 °C. The hydrogen penetration rate was measured with a mass flow meter. Prior to each hydrogen penetration test, the membranes were first confirmed to be with no obvious defects via helium leakage tests. The selectivity was analyzed via the H_2_/N_2_ flux ratio.

## 4. Conclusions

A unique method for synthesizing a modifier based on non-classic bimetallic pentagonally structured Pd-Pt nanoparticles was developed. This method combined classic and additional tools for adjusting the shape and structure of particles, namely, voltage and current. The obtained particles in the modifier composition had high-energy high-index facets. The modifier had a high density of undercoordinated atoms with high reactivity. It was also found that nanoparticles had unique hollow structures. This can significantly reduce the cost due to the economical use of metal. According to the results of the electrochemical studies, the synthesized bimetallic pentagonally structured Pd-Pt/Pd-Ag modifier showed excellent catalytic activity (up to 60.72 mA cm^−2^), long-term stability, and resistance to CO_ads_ poisoning in the alkaline oxidation reaction of methanol. The achieved results can be caused by both the synergistic effect of the combination of Pd and Pt metals in the modifier composition and the large number of available catalytically active centers, which were present due to the non-classic morphology with high-index facets. Very high hydrogen permeability values were recorded while analyzing the results of the membrane performance of the developed materials. This became possible due to the significant facilitation of surface processes, namely, dissociative adsorption and recombinative desorption for modified membranes. The membrane with the pentagonally structured Pd-Pt/Pd-Ag modifier showed the highest hydrogen permeation flux density up to 27.3 mmol s^−1^ m^−2^. The obtained hydrogen flux density was two times higher than that for the membranes with the classic Pd_black_/Pd-Ag modifier and an order of magnitude higher than that for the unmodified membrane. This result can be explained by the presence of nanoparticles in the Pd-Pt/Pd-Ag composition, which were active toward reactions involving hydrogen, due to their high-energy facets with a high index. The specified morphology with an increased number of chemisorption centers made it possible to significantly increase the working surface of the membrane. A significant increase in the selectivity of the developed Pd-Pt/Pd-Ag membranes was recorded, 3514, because the rate of transcrystalline hydrogen transfer through the membrane increased while the rate of transfer through defects remained unchanged. According to the obtained results, no hysteresis dependence was recorded. The membranes demonstrated stability under pressure gradient conditions, and they maintained fairly high permeability and selectivity values over a long period of time. The results, which were obtained in research, were confirmed by the close correlation of diffusion-selective properties in membrane hydrogen mass transfer processes with catalytic properties in processes of alkaline electrooxidation of methanol. The high performance and unique features of the developed modified membrane materials open up prospects for their further research and extension to a wide range of metal and alloy systems.

## Data Availability

The data presented in this study are available on request from the corresponding author.

## References

[1] Filippova S.P., Yaroslavtsev A.B. (2021). Hydrogen energy: Development prospects and materials. Russ. Chem. Rev..

[2] Mamun A., Kiari M., Sabantina L. (2023). A Recent Review of Electrospun Porous Carbon Nanofiber Mats for Energy Storage and Generation Applications. Membranes.

[3] Perović K., Morović S., Jukić A., Košutić K. (2023). Alternative to Conventional Solutions in the Development of Membranes and Hydrogen Evolution Electrocatalysts for Application in Proton Exchange Membrane Water Electrolysis: A Review. Materials.

[4] Migliari L., Micheletto D., Cocco D. (2023). Performance Analysis of a Diabatic Compressed Air Energy Storage System Fueled with Green Hydrogen. Energies.

[5] Binazadeh M., Mamivand S., Sohrabi R., Taghvaei H., Iulianelli A. (2023). Membrane reactors for hydrogen generation: From single stage to integrated systems. Int. J. Hydrogen Energy.

[6] Nemitallah M.A. (2023). Characteristics of hydrogen separation and methane steam reforming in a Pd-based membrane reactor of shell and tube design. Case Stud. Therm. Eng..

[7] Aditiya H.B., Aziz M. (2021). Prospect of hydrogen energy in Asia-Pacific: A perspective review on techno-socio-economy nexus. Int. J. Hydrogen Energy.

[8] Solomin E., Salah Z., Osintsev K., Aliukov S., Kuskarbekova S., Konchakov V., Olinichenko A., Karelin A., Tarasova T. (2023). Ecological Hydrogen Production and Water Sterilization: An Innovative Approach to the Trigeneration of Renewable Energy Sources for Water Desalination: A Review. Energies.

[9] Zhang L., Xie G., Liu F., Ji H. (2023). High hydrogen selectivity Pd-Ni alloy film hydrogen sensor with hybrid organosilica membranes. J. Alloys Compd..

[10] Xu F., Ma J., Li C., Ma C., Li J., Guan B.-O., Chen K. (2023). Fabry–Pérot Cavities with Suspended Palladium Membranes on Optical Fibers for Highly Sensitive Hydrogen Sensing. Molecules.

[11] Jung W., Chang D. (2023). Deep Reinforcement Learning-Based Energy Management for Liquid Hydrogen-Fueled Hybrid Electric Ship Propulsion System. J. Mar. Sci. Eng..

[12] Parra D., Valverde L., Pino F.J., Patel M.K. (2019). A review on the role, cost and value of hydrogen energy systems for deep decarbonisation.. Renew. Sustain. Energy Rev..

[13] Yin Z., Yang Z., Tong Y., Du M., Mi J., Yu Q., Li S. (2023). Improved sulfur tolerance of Pd–Ru membranes: Influence of H2S concentration and exposure time on the hydrogen flux. Int. J. Hydrogen Energy.

[14] Buonomenna M.G. (2023). Proton-Conducting Ceramic Membranes for the Production of Hydrogen via Decarbonized Heat: Overview and Prospects. Hydrogen.

[15] Bernardo G., Araújo T., Lopes T.S., Sousa J., Mendes A. (2020). Recent advances in membrane technologies for hydrogen purification. Int. J. Hydrogen Energy.

[16] Skolotneva E., Tsygurina K., Mareev S., Melnikova E., Pismenskaya N., Nikonenko V. (2022). High Diffusion Permeability of Anion-Exchange Membranes for Ammonium Chloride: Experiment and Modeling. Int. J. Mol. Sci..

[17] Mareev S., Gorobchenko A., Ivanov D., Anokhin D., Nikonenko V. (2023). Ion and Water Transport in Ion-Exchange Membranes for Power Generation Systems: Guidelines for Modeling. Int. J. Mol. Sci..

[18] Zhang K., Way J.D. (2017). Palladium-copper membranes for hydrogen separation. Sep. Purif. Technol..

[19] Mamivand S., Binazadeh M., Sohrabi R. (2021). Applicability of membrane reactor technology in industrial hydrogen producing reactions: Current effort and future directions. J. Ind. Eng. Chem..

[20] Petriev I.S., Lutsenko I.S., Pushankina P.D., Frolov V.Y., Glazkova Y.S., Malkov T.I., Gladkikh A.M., Otkidach M.A., Sypalo E.B., Baryshev P.M. (2022). Hydrogen Transport through Palladium-Coated Niobium Membranes. Russ. Phys. J..

[21] Lytkina A.A., Orekhova N.V., Ermilova M.M., Petriev I.S., Baryshev M.G., Yaroslavtsev A.B. (2019). Ru–Rh based catalysts for hydrogen production via methanol steam reforming in conventional and membrane reactors. Int. J. Hydrogen Energy.

[22] Chen W.-H., Kuo P.-C., Lin Y.-L. (2019). Evolutionary computation for maximizing CO_2_ and H_2_ separation in multiple-tube palladium-membrane systems. Appl. Energy.

[23] Flanagan T.B., Oates W.A. (1991). The Palladium-Hydrogen System. Annu. Rev. Mater. Sci..

[24] Bosko M.L., Fontana A.D., Tarditi A., Cornaglia L. (2021). Advances in hydrogen selective membranes based on palladium ternary alloys. Int. J. Hydrogen Energy.

[25] Zhu K., Li X., Zhang Y., Zhao X., Liu Z., Guo J. (2022). Tailoring the hydrogen transport properties of highly permeable Nb51W5Ti23Ni21 alloy membrane by Pd substitution. Int. J. Hydrogen Energy.

[26] Alrashed F.S., Paglieri S.N., Alismail Z.S., Khalaf H., Harale A., Overbeek J.P., van Veen H.M., Hakeem A.S. (2021). Steam reforming of simulated pre-reformed naphtha in a PdAu membrane reactor. Int. J. Hydrogen Energy.

[27] Suzuki A., Yukawa H. (2020). Analysis for Reverse Temperature Dependence of Hydrogen Permeability through Pd-X (X = Y, Ho, Ni) Alloy Membranes Based on Hydrogen Chemical Potential. Membranes.

[28] Zhou Q., Luo S., Zhang M., Liao N. (2022). Selective and efficient hydrogen separation of Pd–Au–Ag ternary alloy membrane. Int. J. Hydrogen Energy.

[29] Petriev I.S., Pushankina P.D., Lutsenko I.S., Baryshev M.G. (2021). Anomalous Kinetic Characteristics of Hydrogen Transport through Pd–Cu Membranes Modified by Pentatwinned Flower-Shaped Palladium Nanocrystallites with High-Index Facets. Tech. Phys. Lett..

[30] Petriev I.S., Lutsenko I.S., Voronin K.A., Pushankina P.D., Baryshev M.G. (2020). Hydrogen permeability of surface-modified Pd-Ag membranes at low temperatures. IOP Conf. Ser. Mater. Sci. Eng..

[31] Petriev I., Pushankina P., Bolotin S., Lutsenko I., Kukueva E., Baryshev M. (2021). The influence of modifying nanoflower and nanostar type Pd coatings on low temperature hydrogen permeability through Pd-containing membranes. J. Membr. Sci..

[32] Vicinanza N., Svenum I.-H., Peters T., Bredesen R., Venvik H. (2018). New Insight to the Effects of Heat Treatment in Air on the Permeation Properties of Thin Pd77%Ag23% Membranes. Membranes.

[33] Petriev I., Pushankina P., Shostak N., Baryshev M. (2022). Gas-Transport Characteristics of PdCu–Nb–PdCu Membranes Modified with Nanostructured Palladium Coating. Int. J. Mol. Sci..

[34] Kozmai A., Pismenskaya N., Nikonenko V. (2022). Mathematical Description of the Increase in Selectivity of an Anion-Exchange Membrane Due to Its Modification with a Perfluorosulfonated Ionomer. Int. J. Mol. Sci..

[35] Petriev I., Pushankina P., Glazkova Y., Andreev G., Baryshev M. (2023). Investigation of the Dependence of Electrocatalytic Activity of Copper and Palladium Nanoparticles on Morphology and Shape Formation. Coatings.

[36] Shan H., Wang W., Wang Z., Ge J., Liu Q., Zhang W., Fu Q. (2023). General synthesis of flexible CuO nanoparticles-anchored ZrO2 nanofibrous membranes for catalytic oxidation of tetracycline. J. Chem. Eng..

[37] Shkirskaya S.A., Kononenko N.A., Timofeev S.V. (2022). Structural and Electrotransport Properties of Perfluorinated Sulfocationic Membranes Modified by Silica and Zirconium Hydrophosphate. Membranes.

[38] Basov A., Dzhimak S., Sokolov M., Malyshko V., Moiseev A., Butina E., Elkina A., Baryshev M. (2022). Changes in Number and Antibacterial Activity of Silver Nanoparticles on the Surface of Suture Materials during Cyclic Freezing. Nanomaterials.

[39] Mutalik C., Saukani M., Khafid M., Krisnawati D.I., Widodo, Darmayanti R., Puspitasari B., Cheng T.-M., Kuo T.-R. (2023). Gold-Based Nanostructures for Antibacterial Application. Int. J. Mol. Sci..

[40] Stenina I., Yurova P., Achoh A., Zabolotsky V., Wu L., Yaroslavtsev A. (2023). Improvement of Selectivity of RALEX-CM Membranes via Modification by Ceria with a Functionalized Surface. Polymers.

[41] Ramos-Zúñiga J., Bruna N., Pérez-Donoso J.M. (2023). Toxicity Mechanisms of Copper Nanoparticles and Copper Surfaces on Bacterial Cells and Viruses. Int. J. Mol. Sci..

[42] Sogorb M.A., Candela H., Estévez J., Vilanova E. (2023). Investigation of the Effects of Metallic Nanoparticles on Fertility Outcomes and Endocrine Modification of the Hypothalamic-Pituitary-Gonadal Axis. Int. J. Mol. Sci..

[43] Petriev I.S., Pushankina P.D., Lutsenko I.S., Baryshev M.G. (2021). The influence of a crystallographically atypical pentagonal nanostructured coating on the limiting stage of low-temperature hydrogen transport through Pd–Cu membranes. Dokl. Phys..

[44] Theerthagiri J., Lee S.J., Murthy A.P., Madhavan J., Choi M.Y. (2020). Fundamental aspects and recent advances in transition metal nitrides as electrocatalysts for hydrogen evolution reaction: A review. Curr. Opin. Solid State Mater. Sci..

[45] Bianchini C., Shen P.K. (2009). Palladium-based electrocatalysts for alcohol oxidation in half cells and in direct alcohol fuel cells. Chem. Rev..

[46] Alaqarbeh M., Adil S.F., Ghrear T., Khan M., Bouachrine M., Al-Warthan A. (2023). Recent Progress in the Application of Palladium Nanoparticles: A Review. Catalysts.

[47] Sanap K.K., Mali S.S., Tyagi D., Shirsat A.N., Phapale S.B., Waghmode S.B., Varma S. (2023). Development of a Simple Electroless Method for Depositing Metallic Pt-Pd Nanoparticles over Wire Gauge Support for Removal of Hydrogen in a Nuclear Reactor. Materials.

[48] Pushankina P., Baryshev M., Petriev I. (2022). Synthesis and Study of Palladium Mono- and Bimetallic (with Ag and Pt) Nanoparticles in Catalytic and Membrane Hydrogen Processes. Nanomaterials.

[49] Ruditskiy A., Choi S.I., Peng H.C., Xia Y. (2014). Shape-controlled metal nanocrystals for catalytic applications. MRS Bull..

[50] Liu Q., Rzepka P., Frey H., Tripp J., Beck A., Artiglia L., Ranocchiari M., van Bokhoven J.A. (2022). Sintering behavior of carbon-supported Pt nanoparticles and the effect of surface overcoating. Mater. Today Nano.

[51] Dong K., Dai H., Pu H., Zhang T., Wang Y., Deng Y. (2023). Enhanced electrocatalytic activity and stability of Pd-based bimetallic icosahedral nanoparticles towards alcohol oxidation reactions. Int. J. Hydrogen Energy.

[52] Chen Y., Dai Q., Zhang Q., Huang Y. (2022). Precisely deposited Pd on ZnO (002) facets derived from complex reduction strategy for methanol steam reforming. Int. J. Hydrogen.

[53] Karaman C. (2023). Engineering of N,P,S-Triple doped 3-dimensional graphene architecture: Catalyst-support for “surface-clean” Pd nanoparticles to boost the electrocatalysis of ethanol oxidation reaction. Int. J. Hydrogen Energy.

[54] Pushankina P., Andreev G., Petriev I. (2023). Hydrogen Permeability of Composite Pd–Au/Pd–Cu Membranes and Methods for Their Preparation. Membranes.

[55] Chen S., Yang Y., Zhang M., Ma X., He X., Wang T., Hu X., Mao X. (2022). Bioinspired Pd-Cu Alloy Nanoparticles as Accept Agent for Dye Degradation Performances. Int. J. Mol. Sci..

[56] Rzelewska-Piekut M., Wolańczyk Z., Nowicki M., Regel-Rosocka M. (2023). Precipitation of Pt, Pd, Rh, and Ru Nanoparticles with Non-Precious Metals from Model and Real Multicomponent Solutions. Molecules.

[57] Sengupta T., Moody M., Das M., Reber A.C., Khanna S.N., El-Shall M.S. (2023). Experimental and computational study of the catalytic activity of Pd and PdCu nanoparticle catalysts and clusters supported on reduced graphene oxide and graphene acid for the suzuki cross-coupling reaction. Appl. Catal. A Gen..

[58] Zhang Y., Zhang J., Liu Z., Wu Y., Lv Y., Xie Y., Wang H. (2022). Alloying Iron into Palladium Nanoparticles for an Efficient Catalyst in Acetylene Dicarbonylation. Nanomaterials.

[59] Golubovic J., Rakocevic L., Strbac S. (2022). The Effect of Sulphate and Chloride Palladium Salt Anions on the Morphology of Electrodeposited Pd Nanoparticles and their Catalytic Activity for Oxygen Reduction in Acid and Alkaline Media. Int. J. Electrochem. Sci..

[60] Liu H., Ye F., Yao Q., Cao H., Xie J., Lee J.Y., Yang J. (2014). Stellated Ag-Pt bimetallic nanoparticles: An effective platform for catalytic activity tuning. Sci. Rep..

[61] Sikora E., Koncz-Horváth D., Muránszky G., Kristály F., Fiser B., Viskolcz B., Vanyorek L. (2021). Development of Nickel- and Magnetite-Promoted Carbonized Cellulose Bead-Supported Bimetallic Pd–Pt Catalysts for Hydrogenation of Chlorate Ions in Aqueous Solution. Int. J. Mol. Sci..

[62] Lei C., Zhou Z., Chen W., Xie J., Huang B. (2022). Polypyrrole supported Pd/Fe bimetallic nanoparticles with enhanced catalytic activity for simultaneous removal of 4-chlorophenol and Cr(VI). Sci. Total Environ..

[63] Asgar Pour Z., Abduljawad M.M., Alassmy Y.A., Alnafisah M.S., El Hariri El Nokab M., Van Steenberge P.H.M., Sebakhy K.O. (2023). Synergistic Catalytic Effects of Alloys of Noble Metal Nanoparticles Supported on Two Different Supports: Crystalline Zeolite Sn-Beta and Carbon Nanotubes for Glycerol Conversion to Methyl Lactate. Catalysts.

[64] Gulbagça F., Aygun A., Altuner E.E., Bekmezci M., Gur T., Sen F., Karimi-Maleh H., Zare N., Karimi F., Vasseghian Y. (2022). Facile bio-fabrication of Pd-Ag bimetallic nanoparticles and its performance in catalytic and pharmaceutical applications: Hydrogen production and in-vitro antibacterial, anticancer activities, and model development. Chem. Eng. Res. Des..

[65] Paz-Borbón L.O., Johnston R.L., Barcaro G., Fortunelli A. (2008). Structural motifs, mixing, and segregation effects in 38-atom binary clusters. J. Chem. Phys..

[66] Jung H., King M.E., Personick M.L. (2019). Strategic synergy: Advances in the shape control of bimetallic nanoparticles with dilute alloyed surfaces. Curr. Opin. Colloid Interface Sci..

[67] Shah M.A. (2012). Growth of uniform nanoparticles of platinum by an economical approach at relatively low temperature. Sci. Iran..

[68] Walbrück K., Kuellmer F., Witzleben S., Guenther K. (2019). Synthesis and Characterization of PVP-Stabilized Palladium Nanoparticles by XRD, SAXS, SP-ICP-MS, and SEM. J. Nanomater..

[69] Yang Y., Cao Y., Yang L., Huang Z., Long N.V. (2018). Synthesis of Pt–Pd Bimetallic Porous Nanostructures as Electrocatalysts for the Methanol Oxidation Reaction. Nanomaterials.

[70] Ghosh S., Sahu R.K., Raj C.R. (2012). Pt–Pd alloy nanoparticle-decoratedcarbon nanotubes: A durable andmethanol tolerant oxygen reductionelectrocatalyst. Nanotechnology.

[71] Gutkin M.Y., Kolesnikova A.L., Yasnikov I.S., Vikarchuk A.A., Aifantis E.C., Romanov A.E. (2018). Fracture of hollow multiply-twinned particles under chemical etching. Eur. J. Mech. A Solids.

[72] Yousaf A.B., Khan R., Imran M., Fernandez C., Yuan C.-Z., Song L. (2016). Synergistic Electronic Pull of Graphene Oxide Supported Pd Nanoparticles on Enhancing Catalytic Activity of Electro Deposited Pt Nanoparticles for Methanol Oxidation Reaction. Int. J. Electrochem. Sci..

[73] Kim S.-M., Lee Y.-J., Kim J.-W., Lee S.-Y. (2014). Facile synthesis of Pt–Pd bimetallic nanoparticles by plasma discharge in liquid and their electrocatalytic activity toward methanol oxidation in alkaline media. Thin Solid Film..

[74] Zhan F., Bian T., Zhao W., Zhang H., Jin M., Yang D. (2014). Facile synthesis of Pd–Pt alloy concave nanocubes with high-index facets as electrocatalysts for methanol oxidation. CrystEngComm.

[75] Hanifah M.F.R., Jaafar J., Othman M.H.D., Ismail A.F., Rahman M.A., Yusof N., Aziz F., Rahman N.A. (2019). One-pot synthesis of efficient reduced graphene oxide supported binary Pt-Pd alloy nanoparticles as superior electro-catalyst and its electro-catalytic performance toward methanol electro-oxidation reaction in direct methanol fuel cell. J. Alloys Compd..

[76] Woo S., Lee J., Park S.-K., Kim H., Chung T.D., Piao Y. (2015). Electrochemical codeposition of Pt/graphene catalyst for improved methanol oxidation. Curr. Appl. Phys..

[77] Chen D., Zhao Y., Fan Y., Wang W., Li X., Peng X., Wang X., Tian J. (2014). Preparation and characterization of core–shell-like PbPt nanoparticles electro-catalyst supported on graphene for methanol oxidation. Int. J. Hydrogen Energy.

[78] Zhang Y., Chang G., Shu H., Oyama M., Liu X., He Y. (2014). Synthesis of Pt–Pd bimetallic nanoparticles anchored on graphene for highly active methanol electro-oxidation. J. Power Sources.

[79] Kim T.-W., Lee E.-H., Byun S., Seo D.-W., Hwang H.-J., Yoon H.-C., Kim H., Ryi S.-K. (2022). Highly selective Pd composite membrane on porous metal support for high-purity hydrogen production through effective ammonia decomposition. Energy.

[80] Iulianelli A., Ghasemzadeh K., Marelli M., Evangelisti C. (2019). A supported Pd-Cu/Al2O3 membrane from solvated metal atoms for hydrogen separation/purification. Fuel Process. Technol..

[81] Yin Z., Yang Z., Du M., Mi B.J., Hao L., Tong Y., Feng Y., Li S. (2022). Effect of annealing process on the hydrogen permeation through Pd–Ru membrane. J. Membr. Sci..

[82] Petriev I.S., Pushankina P.D., Andreev G.A. (2023). Investigation of Low-Temperature Hydrogen Permeability of Surface Modified Pd–Cu Membranes. Membr. Membr. Technol..

